# *In vitro* functional validation of anti-CD19 chimeric antigen receptor T cells expressing lysine-specific demethylase 1 short hairpin RNA for the treatment of diffuse large B cell lymphoma

**DOI:** 10.3389/fimmu.2024.1521778

**Published:** 2025-01-13

**Authors:** Zhi Guo, Mingxin He, Ning Liu, Yiqing Yang, Rui Sun, Jianxun Wang, Qiang Wang

**Affiliations:** ^1^ Institute of Infection, Immunology and Tumor Microenvironment, Hubei Province Key Laboratory of Occupational Hazard Identification and Control, School of Medicine, Wuhan University of Science and Technology, Wuhan, China; ^2^ Department of Hematology, The 6th Affiliated Hospital of Shenzhen University Health Science Center, Shenzhen, China; ^3^ Shenzhen Cell Valley Biomedical Co., LTD, Shenzhen, China

**Keywords:** LSD1 shRNA, diffuse large B cell lymphoma (DLBCL), anti-CD19 CAR-T cells, *in vitro* functional validation, cell therapy

## Abstract

**Background:**

Chimeric antigen receptor T (CAR-T) cell therapy is more effective in relapsed or refractory diffuse large B cell lymphoma (DLBCL) than other therapies, but a high proportion of patients relapse after CAR-T cell therapy owing to antigen escape, limited persistence of CAR-T cells, and immunosuppression in the tumor microenvironment. CAR-T cell exhaustion is a major cause of relapse. Epigenetic modifications can regulate T cell activation, maturation and depletion; they can be applied to reduce T cell depletion, improve infiltration, and promote memory phenotype formation to reduce relapse after CAR-T cell therapy.

**Purpose:**

We propose to develop and validate *in vitro* the function of novel CAR-T cells for the treatment of DLBCL, which simultaneously express an anti-CD19 CAR with lysine-specific demethylase 1 (LSD1) short hairpin (sh)RNA to prevent depletion and prolong the survival of CAR-T cells.

**Methods:**

We designed an shRNA sequence targeting LSD1 mRNA, and created a vector with the following elements: the U6 promoter driving expression of the LSD1 shRNA sequence, the EF1a promoter driving a second-generation anti-CD19 CAR sequence encoding an anti-CD19 single-chain variable fragment (FMC63), the CD8 hinge and transmembrane structural domains, the CD28 co-stimulatory structural domain, and the CD3ζ-activating structural domain. The MFG-LSD1 shRNA anti-CD19 CAR plasmid was first constructed, then packaged in retroviral vectors and transduced into human primary peripheral blood mononuclear cell-derived T cells to generate the corresponding CAR-T cells. We examined by flow cytometry the efficiency of two CAR-T cells in killing U-2932 cells (a human DLBCL line) upon co-culture with RNAU6 anti-CD19 CAR-T cells or LSD1 shRNA anti-CD19 CAR-T cells. We analyzed Ki-67 staining of the CAR-T cells by flow cytometry on days 0, 5, and 10, and counted the cells to assess expansion. We also used flow cytometry to detect the central memory T cell (TCM) proportion.

**Results:**

We detected the expression of the CAR in the CAR-T cells by flow cytometry, and observed transduction rates of 31.5% for RNAU6 anti-CD19 CAR-T cells and 60.7% for LSD1 shRNA anti-CD19 CAR-T cells. The killing efficiency of LSD1 shRNA anti-CD19 CAR-T cells was significantly higher than that of RNAU6 anti-CD19 CAR-T cells at the low effector target ratio. We further found that LSD1 shRNA anti-CD19 CAR-T cells secreted more IFN-γ and granzyme B than RNAU6 anti-CD19 CAR-T cells. CAR-T cells proliferated after U-2932 cell stimulation and were able to sustain proliferation. After stimulation via U-2932 cell co-culture, both RNAU6 anti-CD19 CAR-T and LSD1 shRNA anti-CD19 CAR-T populations had increased proportions of cells with the TCM phenotype, with a higher percentage among LSD1 shRNA anti-CD19 CAR-T cells.

**Conclusion:**

We developed a novel, feasible CD19-LSD1 shRNA CAR-T cell strategy for the treatment of DLBCL. Our *in vitro* assay results showed that LSD1 shRNA anti-CD19 CAR-T cells more effectively killed target cells than RNAU6 anti-CD19 CAR-T cells, and developed a higher proportion of TCM phenotype cells. LSD1 shRNA anti-CD19 CAR-T cells may represent a potential treatment for DLBCL.

## 1 Introduction

Chimeric antigen receptor T (CAR-T) cell therapy has been a major breakthrough in the treatment of diffuse large B cell lymphoma (DLBCL). The transformation of T cells through genetic engineering into novel killer cells that target CD19 is highly specific, efficient, and clinically effective. Using this system, the recognition of tumor antigens is not dependent on major histocompatibility complex and the system enhances the targeted killing and persistence of effector T cells (1–4). CAR-T cell therapy targeting the B lymphocyte surface antigen CD19 is most effective for relapsed and refractory lymphomas (5–8), and several CAR-T products are currently approved as second-line therapeutic regimens for relapsed and refractory large B cell lymphomas, including DLBCL (9–12). In addition, in patients with relapsed or refractory large B cell lymphoma, second-line treatment with CAR-T cells significantly prolongs overall survival compared with standard therapy (13, 14). Despite the unprecedented success of CAR-T cell therapy in the treatment of relapsed and refractory B cell tumors, a notable percentage of patients relapse after CAR-T cell therapy owing to antigenic escape, the limited persistence of CAR-T cells, and immunosuppression in the tumor microenvironment (15).

CAR-T cell exhaustion is one of the major causes of relapse after CAR-T cell therapy. In recent years, studies have demonstrated the ability of epigenetic mechanisms to regulate T cell activation, maturation, and depletion. Genomic or DNA modifications, histone modifications, and non-coding RNA modifications can increase cell persistence and survival, reduce T cell depletion, improve T cell infiltration, and promote memory phenotype formation to overcome CAR-T cell therapy limitations (16). Lysine-specific demethylase 1 (LSD1, also known as KDM1A, AOF2, and BHC110) is ubiquitously overexpressed in diverse cancers, and abrogation of LSD1 expression inhibits the proliferation, invasion, and migration of cancer cells (17). LSD1 specifically demethylates histone lysine residues H3K4me1/2 and H3K9me1/2, and plays an important role in gene expression (18). Targeting histone demethylase LSD1 with chemical inhibitors can remodel the epigenome of activated and expanded CD8^+^ T cells *in vitro* and enhance their anti-tumor efficacy (19). Recent studies have shown that LSD1 inhibition combined with TGF-β and PD-1 blockade significantly increases CD8^+^ T cell infiltration and cytotoxicity. LSD1 contributes to the regulation of Th1 cell differentiation, and pharmacological inhibition of LSD1 induces IFN-γ production by Th1 cells, suggesting that LSD1 inhibition may be a potential strategy to improve Th1 cell differentiation and cytotoxic T cell immune function (20). LSD1 deficiency enhances tumor immunogenicity and T cell infiltration, so LSD1 inhibition combined with PD-(L)1 blockade could be a novel cancer treatment strategy. Currently, many LSD1 inhibitors are under clinical evaluation for cancer therapy (21). Given the important role of LSD1 in T cells in the tumor microenvironment, we investigated the effect of knocking down LSD1 expression in T cells on CAR-T cell function. In this study, we developed a strategy for generating CAR-T cells that simultaneously express an anti-CD19 CAR and LSD1 shRNA, with the aim of targeting CD19 and improving the efficacy of CAR-T cell therapy by preventing T cell depletion.

## 2 Materials and methods

### 2.1 Experimental materials

We used the cell lines U-2932 (human diffuse large B lymphoma), 293T (human embryonic kidney cells), Phoenix-ECO (human retroviral packaging cell line), and PG13 (retroviral-encapsulated TK-NIH3T3 cells); the Phoenix-ECO and PG13 cells were obtained from the American Type Culture Collection, and the U-2932 cell line was purchased from Genjing Bio. All cell lines were verified by short tandem repeat testing by the providers. We used the plasmids MFG-RNAU6 anti-CD19 CAR (CD19 CAR retroviral vector) and MFG-LSD1 shRNA anti-CD19 CAR (LSD1 knockdown and CD19 CAR co-expression retroviral vector). The main experimental reagents included OKT3 and IL-2 purchased from Yiqiao Shenzhou, RetroNectin purchased from TAKARA, polybrene purchased from MCE, diethyl pyrocarbonate-treated water purchased from Biosharp Biologicals, aminobenzylpenicillin purchased from Abcam, a Plasmid Extraction Kit purchased from Kangwei Century, and an Annexin V-FITC/PI Apoptosis Kit purchased from Prygene. The experiments were conducted with biological safety cabinets and incubators from ESCO, general optical microscopes and fluorescence microscopes from ZEISS, centrifuges from ThermoFisher, flow cytometers from BD, thermocyclers from Biorad, a qPCR unit from Roche, and water purifiers from Milli-Q. We used commercially available antibodies, including monoclonal anti-(G4S)n(B02H1) (APC); FITC-Labeled Human CD19 (20–291), Protein, Fc Tag DMF Filed; and antibodies against CD45RO, CD62L, and CD3.

### 2.2 Cell culture methods

We cultured the 293T, Phoenix-ECO, and PG13 cells in Dulbecco’s Modified Eagle Medium (Gibco, USA) supplemented with 10% fetal bovine serum. U-2932 cells stably expressing firefly luciferase were cultured in RPMI 1640 medium (Gibco) containing 10% fetal bovine serum. When culturing 293T cells—which are relatively weakly adherent in suboptimal conditions—an obvious patchy detachment phenomenon was occasionally observed. If the shedding was not severe the cells were placed back into the medium as soon as possible to continue the culture. In cases of large-scale shedding, the cells were collected, re-digested, and dispersed before inoculation.

### 2.3 Construction, packaging, and biological titer assays of retroviral vectors

The vector consisted of a U6 promoter followed by an LSD1 shRNA sequence, an EF1a promoter followed by a second-generation anti-CD19 CAR sequence encoding an anti-CD19 single-chain variable fragment (FMC63), a CD8 hinge and transmembrane structural domain, a CD28 co-stimulatory structural domain, and a CD3ζ-activating structural domain. The heavy and light chains (FMC63) were separated by a (G4S)3 linker. The G4S tag was used to detect transduction efficiency. The LSD1 shRNA sequence was 5’-CCGGGCTCCAATACTGTTGGCACTACTCGAGTAGTGCCAACAGTATTGGAGCTTTTTG-3’. For retroviral vector packaging, we first used Phoenix-ECO cells to produce a pro-retroviral vector, and then infected PG13 cells with this pro-retroviral vector to form a stable virulence-producing retroviral vector-packaging cell line. The stably transfected PG13 cell line was used to generate retroviral vectors for transducing activated human T cells. We detected the biological titers of the viral vectors by flow cytometry. First, 293T cells were seeded in 24-well plates at a density of 1.5 × 10^5^ cells/wells. Then, the retroviral vectors were diluted to different ratios and added to the 24-well plate, which was then subjected to centrifugation at 1,460 *g* and 32°C for 1 h. After 24 h of transduction, the medium in the well plates was replaced with complete medium without retroviral vectors. After a further 48 h, the cells in the well plates were digested, stained, and assayed on a flow cytometer to calculate the biological titers of the retroviral vectors.

### 2.4 Recovery of peripheral blood mononuclear cells and T cell activation

We quickly transferred the tubes containing cells from the liquid nitrogen tank to a pre-warmed 37°C water bath; after thawing with shaking and confirming complete dissolution, we used a pipette to slowly transfer the cells dropwise to a centrifuge tube containing 9 mL of X-VIVO with 5% fetal bovine serum (complete culture medium). The cells were centrifuged at 300 g for 10 min, after which the supernatant was removed and 1 mL of the complete medium was used to resuspend the cells for counting. Using the complete medium, the cell density was adjusted to 2 × 10^6^/mL. We added OKT3 to a final concentration of 100 ng/mL and IL-2 to a final concentration of 500 U/mL. We then seeded the cells in six-well plates (2 mL cell suspension/well). The plates were labeled with the cell name and time, then incubated at 37°C in 5% CO_2_.

### 2.5 T cell transduction and culture

After 48 h of stimulation, the T cells were removed from the incubator and observed under the microscope for T cell cluster formation and to confirm the conditions were appropriate for retroviral transduction. One day before transduction, we coated a plate with RetroNectin by adding 1 mL of diluted RetroNectin (10 μg/mL) to one well of a 12-well plate (non-tissue culture-treated), mixing gently, and marking the corresponding position in the plate. We sealed the 12-well plate with a sealing film and excluded light by wrapping in foil, then stored the plate at 4°C overnight; the RetroNectin solution was aspirated on day 2 of transduction, and the plate was washed with 1 mL of phosphate-buffered saline (PBS) while the frozen viral vector was thawed at 4°C. We removed the PBS and add 1 mL of retroviral vector, then centrifuged the plate at 1,460 *g* at a temperature of 32°C for 1 h. We resuspended the T cells and used 10 μL of the cell samples for counting. We transferred 0.4 × 10^6^ cells/well into a 1.5 mL centrifuge tube for transduction. The T cells were centrifuged at 400 g for 5 min, after which the medium was discarded and the cells were resuspended with 1 mL of retroviral vector and mixed by adding polybrene at a final concentration of 6 μg/μL. The centrifuged 12-well plate was removed and the retroviral vector was removed. The cell suspension was added dropwise to a 12-well plate coated with RetroNectin. The 12-well plates were centrifuged at 1,460 *g* for 1 h at 32°C. After centrifugation, the 12-well plates were returned to the 37°C incubator and incubated for at least 2 h. We collected the T cell suspension from the wells and immediately added 500 μL of retroviral vector to the well plates. Next, we centrifuged the T cell suspension at 400 g for 5 min, then removed the cell supernatant, added 500 μL of retroviral vector to resuspend the cells, added polybrene, and mixed the cells before transferring to 12-well plates. The plates were centrifuged at 1,460 *g* for 1 h at 32°C. After centrifugation, the cells were returned to the 37°C incubator and incubated for at least 2 h. We then collected the cell suspensions from the wells, centrifuged at 400 g for 5 min, discarded the supernatant, and resuspended the cells in fresh T cell complete medium (1 mL/well). We added IL-2 (final concentration 500 U/mL) and assayed the T cell transduction efficiency after 48 h.

### 2.6 Flow assay for T cell retroviral transduction efficiency

CAR-T cells were removed from the incubator, mixed in their culture supernatant, and sampled for cell viability and density. We transferred 5×10^5^ CAR-T cells to a 1.5 mL centrifuge tube and centrifuged at 400 g for 5 min. Next, we discarded the supernatants, add 1 mL of PBS to wash the cells once, and then centrifuged at 400 g for 5 min. We discarded the supernatants, then added 100 µL of PBS to resuspend the cells, added 5 µL FITC-Labeled Human CD19 (20–291) Protein Antibody, mixed well, and incubated for 30 min at 4°C in a refrigerator, protected from light. After the incubation, we centrifuged the cells at 400 g for 5min, discarded the supernatant and washed once with 1 mL of PBS, centrifuged again at 400 g for 5 min, and then discarded the supernatant. We resuspended the cells in 300 µL of PBS and assayed for transduction by flow cytometry.

### 2.7 CAR-T cell-initiated *in vitro* apoptosis assay

#### 2.7.1 Cell preparation

We co-cultured the effector cells (pan-T cells or CAR-T cells) with the target cells (tumor cells) at ratios of 4:1, 2:1, 1:1, and 0.5:1. The target cells were first diluted to 4 × 10^5^ cells/mL and then 100 µL of target cell suspension was added to each well (4 × 10^4^ target cells/well). The effector cells were diluted proportionally in X-VIVO complete medium containing 500 U/mL IL-2. We added 100 µL of each diluted effector cell suspension to the appropriate well. Then, the 96-well plate was placed in a cell incubator for 16 h, after which the cells were collected. We used flow cytometry to detect the target cell killing efficiency of the effector cells.

#### 2.7.2 *In vitro* killing efficiency by flow cytometry

The samples to be tested were grouped together. Target cells that were not co-cultured with effector cells were used as controls, and target cell apoptosis was detected by flow cytometry. The collected cells were transferred to a 1.5 mL centrifuge tube and centrifuged at 400 g for 5 min. Then, the supernatants were carefully removed, the cells were washed by adding 1 mL of PBS, centrifuged again at 400 g for 5 min, and the supernatants were discarded. This procedure was repeated to wash the cells twice. We diluted the binding buffer with deionized water at 1:10, and resuspended the cellular precipitate by adding 250 µL of binding buffer to each sample and adjusting to a concentration of 1 × 10^6^/mL. Then, we transferred 100 µL of cell suspension to a 5 mL flow tube and added 5 µL of FITC-labeled Annexin-V Apoptosis Detection Reagent and 10 µL of propidium iodide solution. The suspensions were mixed well and incubated at room temperature away from light for 15 min. Finally, we added 400 µL of PBS to the reaction tube and collected and analyzed the data using a flow cytometer.

### 2.8 CAR-T cell cytokine secretion assay

The effector cells and target cells were co-cultured for 16 h at a ratio of 2:1, and then the supernatant was collected for a cytometric bead array assay. We diluted the supernatant 2-fold or 10-fold with PBS, added 25 μL of assay buffer to each well, transferred 25 μL of standards or samples to the wells, and shook the capture microspheres for 30 s before adding 25 μL to each well. After the sealing film was attached, the plates were incubated at room temperature while being shaken in a shaker at 500 rpm for 2 h, shielded from light. We then centrifuged the plates at 250 g for 5 min, gently discarded the supernatant, and snapped the plate onto a piece of paper to aspirate the liquid. Next, we added 200 μL of 1× wash solution to each well, incubated for 1 min, and then discarded the supernatant after centrifuging at 250 g for 5 min. We added 25 μL of detection antibody to each well, sealed, and incubated at room temperature with shaking in a shaker at 500 rpm for 1 h, shielded from light. After incubation, we added 25 μL of PE-labeled streptavidin, sealed the plate, and incubated at room temperature with shaking in a shaker at 500 rpm for 30 min. Then, we centrifuged at 250 g for 5 min, and discarded the supernatant. We next added 200 μL of 1× wash solution to each well, incubated for 1 min, centrifuged at 250 g for 5 min, discarded the supernatant, and added 150 μL of 1× wash solution to each well to resuspend the microspheres and transfer into tubes for detection. Finally, we performed a flow assay for TNF-α, IFN-γ, and granzyme B secretion.

### 2.9 CAR-T cell proliferation assay *in vitro*

Effector and target cells were co-cultured at a ratio of 2:1 (5×10^5^ effector cells to 2.5×10^5^ target cells), inoculated in 12-well culture plates, and incubated for 0, 5, and 10 d. We prepared 70% ethanol, which we cooled to 4°C. After the incubation period, we prepared the target cells by washing twice with PBS, centrifuging at 350 g for 5 min, discarding the supernatant, and vortexing the cells; we added 3 mL of pre-cooled 70% ethanol dropwise into the cell solution while vortexing, continued vortexing for 30 s, and then incubated the cells for 1 h at −20°C. We washed the cells 3× with cell staining buffer, and resuspended them in cell staining buffer at a concentration at 0.5–10×10^6^ cells/mL. An appropriate amount of Ki-67 antibody was added to 100 μL of cells, which were incubated for 30 min at room temperature away from light. We washed twice with cell staining buffer, then resuspended the cells in 0.5 mL of cell staining buffer and analyzed Ki-67 staining by flow cytometry.

### 2.10 CAR-T cell TCM ratio assay

Effector and target cells were co-cultured at a ratio of 2:1 (5×10^5^ effector cells and 2.5×10^5^ target cells) and inoculated in 12-well culture plates. We collected the cells after incubation for 0, 5, and 10 d. After incubation, we used CD45RO and CD62L antibodies to differentiate between memory and effector T cell populations; TCM were defined as expressing CD45RO and CD62L.

### 2.11 Statistical analysis

Statistical analyses were performed using GraphPad Prism8 software. The data are shown as the mean ± standard deviation. For comparisons between two groups, the unpaired Student’s t-test was performed. All experiments were performed at least three times to establish reproducibility using at least independent donors. Differences for which p < 0.05 were considered statistically significant.

## 3 Results

### 3.1 Successful construction of LSD1 shRNA anti-CD19 CAR-T cells and shRNA downregulation of LSD1 mRNA expression

**Figure 1:**
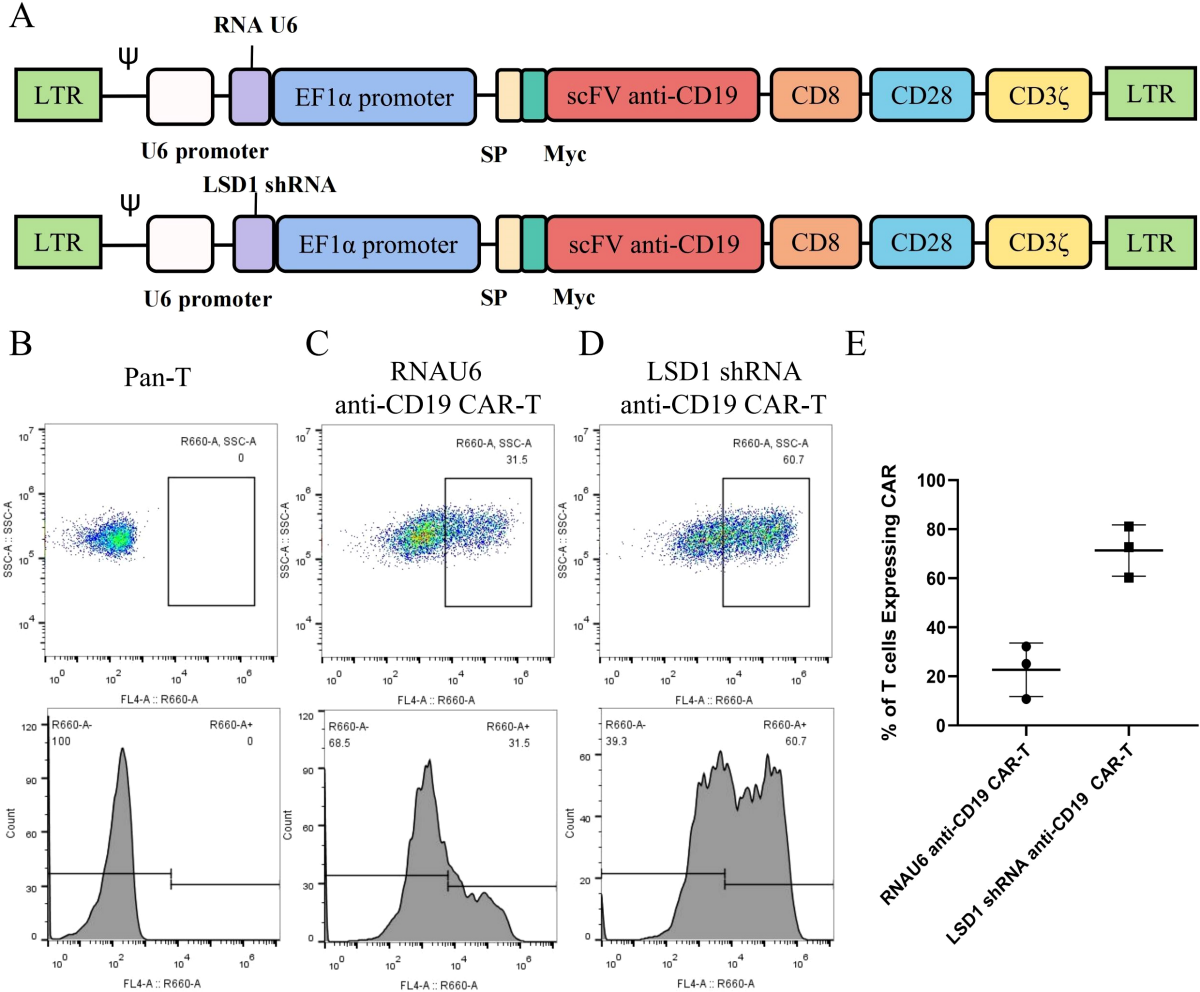
Construction and expression of the anti-CD19 chimeric antigen receptor. **(A)** Schematic diagram of the structure of the anti-CD19 chimeric antigen receptor (CAR). **(B)** Untransduced human primary peripheral blood mononuclear cell-derived T cells. **(C)** RNAU6 anti-CD19 CAR-T cell transduction efficiency. **(D)** LSD1 short hairpin (sh)RNA anti-CD19 CAR-T cell transduction efficiency. **(E)** Transduction efficiency of human T cells with anti-CD19 CAR retroviral vectors analyzed by flow cytometry (n = 3 donors).

**Figure 2:**
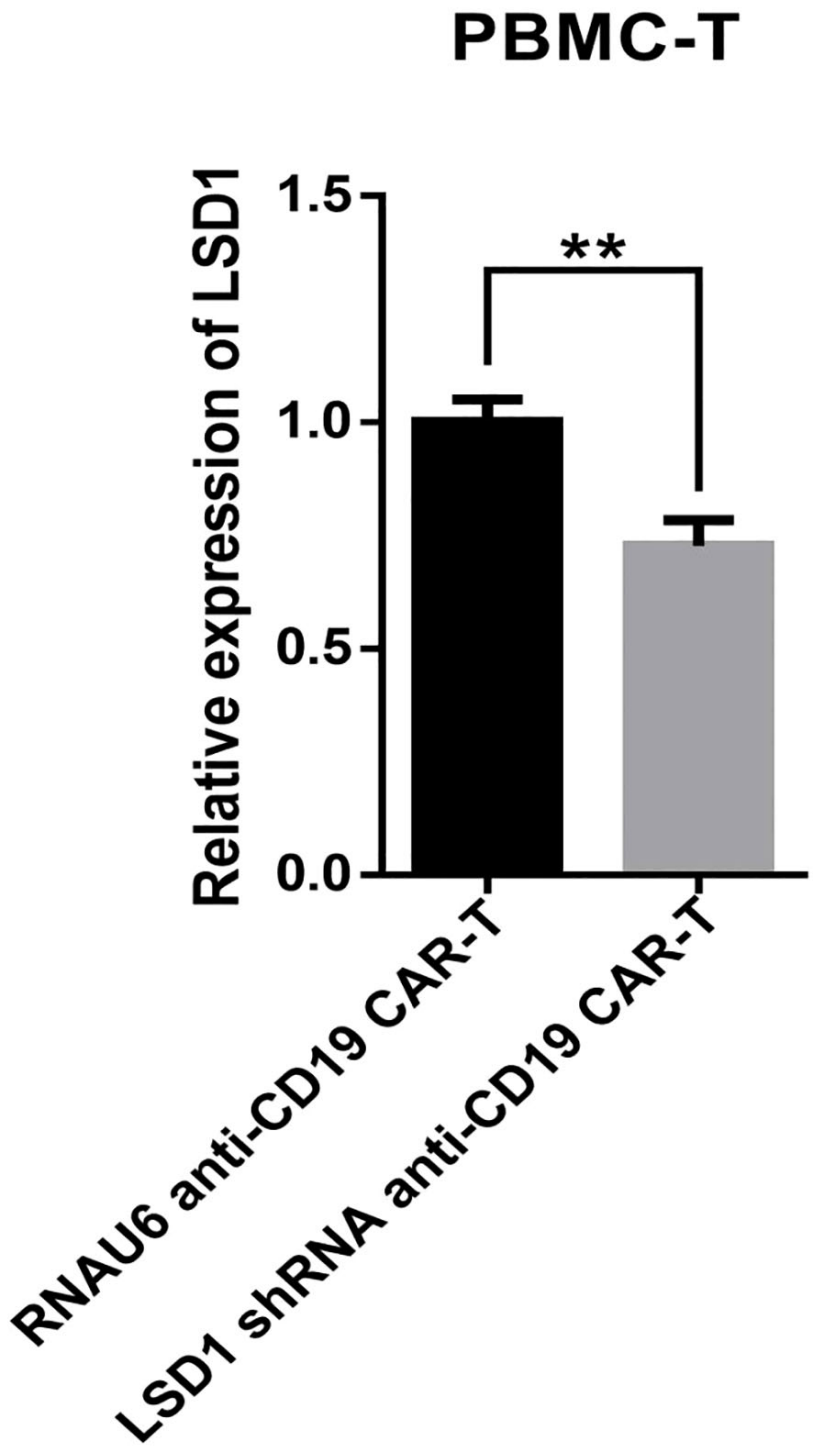
Quantitative reverse transcription polymerase chain reaction-based detection of LSD1 mRNA expression levels (n = 3 donors). Values are expressed as the mean ± standard deviation. The differences were assessed with the unpaired t-test. **p < 0.01.

**Figure 3:**
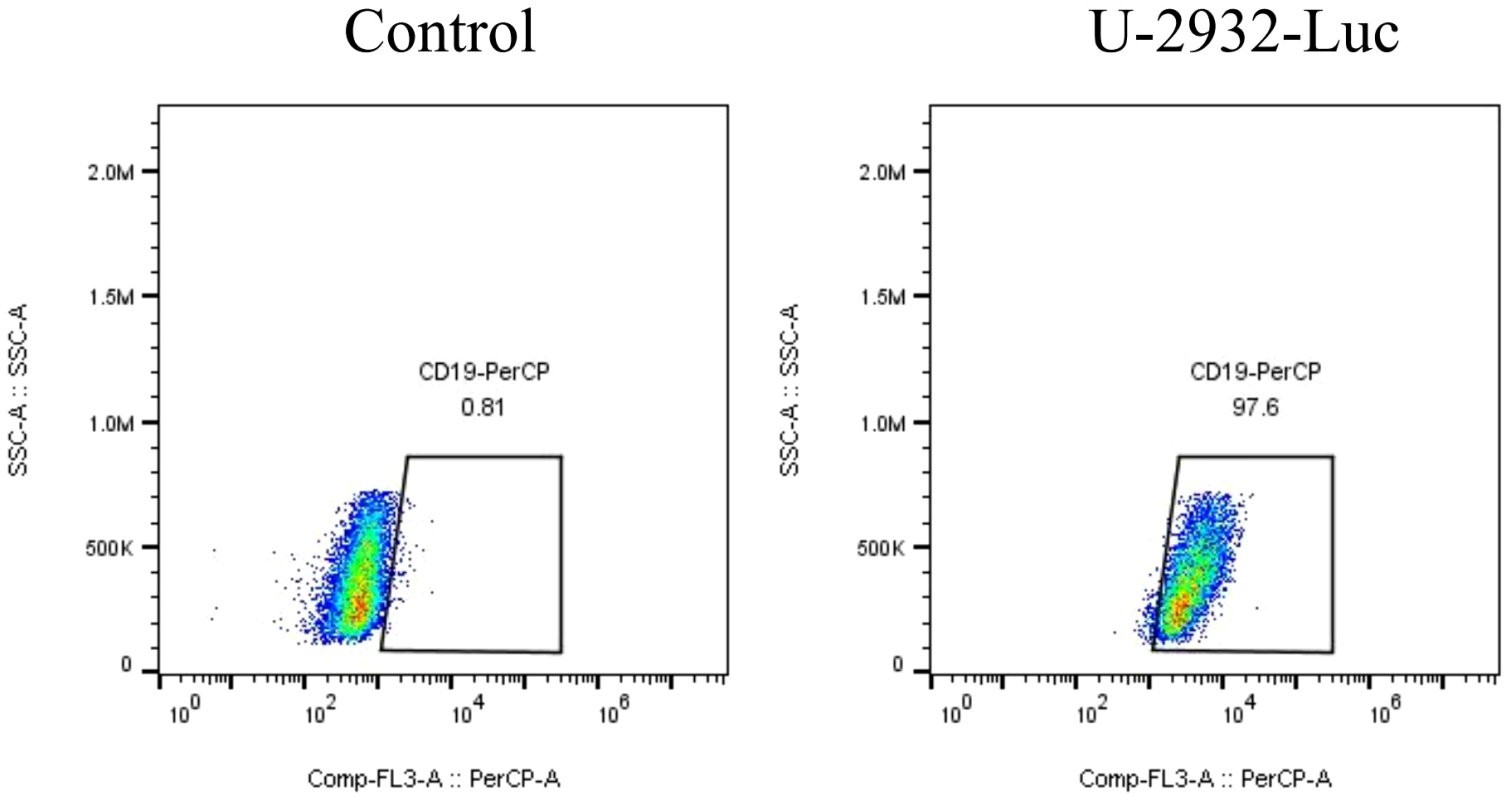
CD19 expression level in U-2932 cells.

We developed a strategy to generate CAR-T cells that simultaneously express an anti-CD19 CAR with LSD1 shRNA. We designed an shRNA sequence targeting LSD1 mRNA. The vector consists of a U6 promoter followed by an LSD1 shRNA sequence, an EF1a promoter followed by a second-generation anti-CD19 CAR sequence encoding an anti-CD19 scFv (FMC63), a CD8 hinge and transmembrane structural domain, a CD28 co-stimulatory structural domain, and a CD3ζ-activating structural domain (**Figure 1A**). After thawing healthy human peripheral blood mononuclear cells, we added 100 ng/mL OKT3 and 500 U/mL IL-2 to activate and expand CD3^+^ T cells; we transduced the modified peripheral blood mononuclear cell-derived T cells with retroviral vectors after 48 h. Then, after 48 h of transduction, we detected CAR expression in the CAR-T cells using flow cytometry. The transduction positivity rate for RNAU6 anti-CD19 CAR-T cells was 31.5%, and that for the LSD1 shRNA anti-CD19 CAR-T cells was 60.7% (**Figures 1B–E**). Downregulation of LSD1 mRNA expression by the shRNA in human T cells was confirmed with quantitative reverse transcription polymerase chain reaction experiments (**Figure 2**). In the validation assay for the killing ability of LSD1 shRNA anti-CD19 CAR-T cells *in vitro*, we confirmed the high CD19 expression levels of the U-2932 target cells (**Figure 3**).

### 3.2 CD19-LSD1 shRNA CAR-T cells effectively killed U-2932 cells

**Figure 4:**
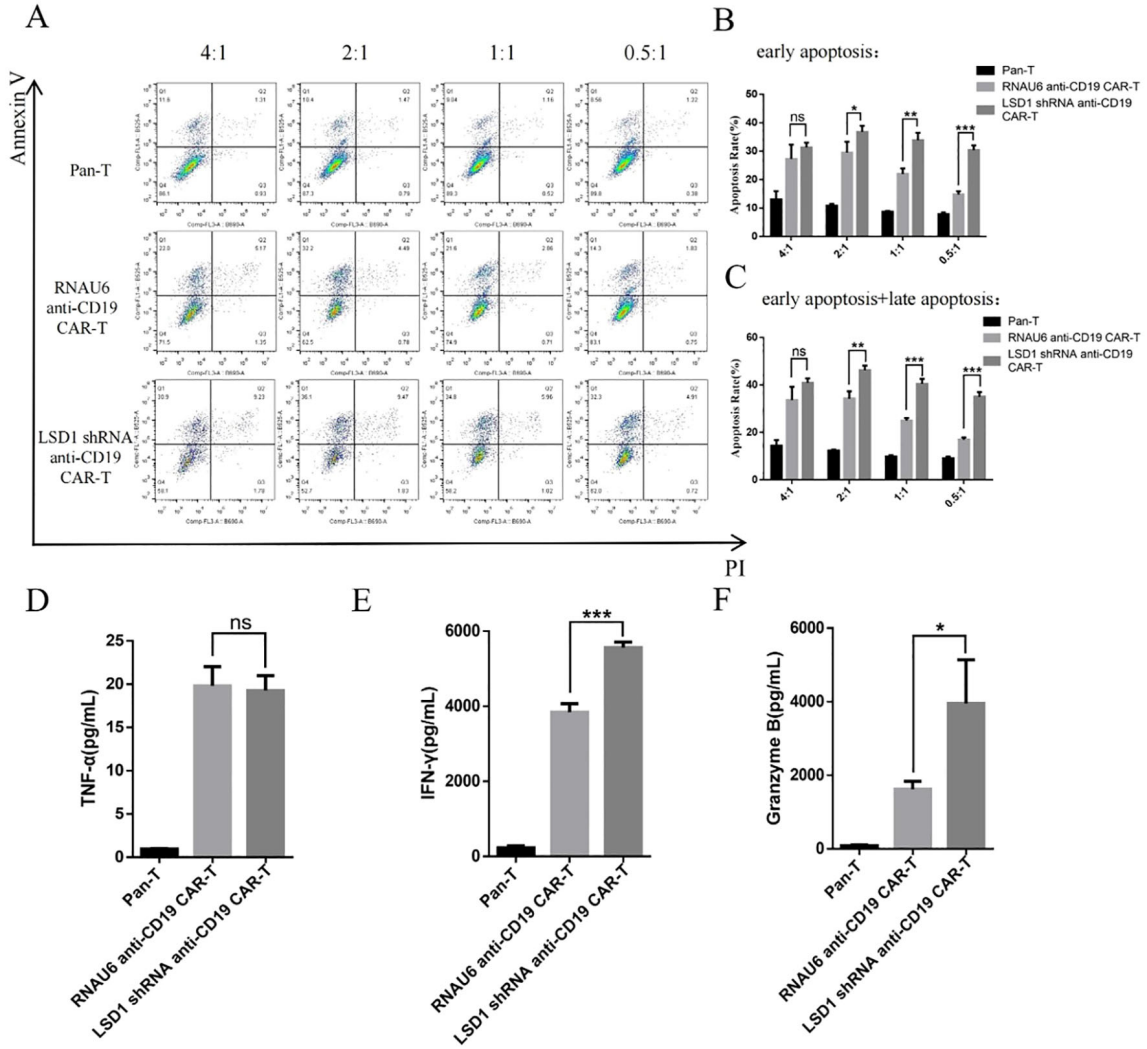
CAR-T cell *in vitro* killing assay. **(A)** Representative images of the flow cytometric apoptosis assay. LSD1 shRNA anti-CD19 CAR-T cells were co-cultured with U-2932 cells over a effector target ratio gradient for 16 h. The apoptosis signals (Annexin V/propidium iodide [PI]) from target cells were measured by flow cytometry. **(B)** Annexin-V^+^PI^−^: early apoptosis, **(C)** Annexin-V^+^PI^−^: early apoptosis, and Annexin-V^+^PI^+^: late apoptosis. **(D)** TNF-α secretion. **(E)** IFN-γ secretion. **(F)** Granzyme B secretion. The levels of TNF-α **(D)**, IFN-γ **(E)**, and granzyme B **(F)** secreted by CAR-T cells after co-culture with U-2932 cells at an effector target ratio of 2:1 for 16 h, detected by cytometric bead array. Values are expressed as the mean ± standard deviation. The unpaired t-test was performed to compare between the groups. ***p < 0.001; **p < 0.01; *p < 0.05; and ns, *p* > 0.05 compared with RNAU6 anti-CD19 CAR-T cells.

**Figure 5:**
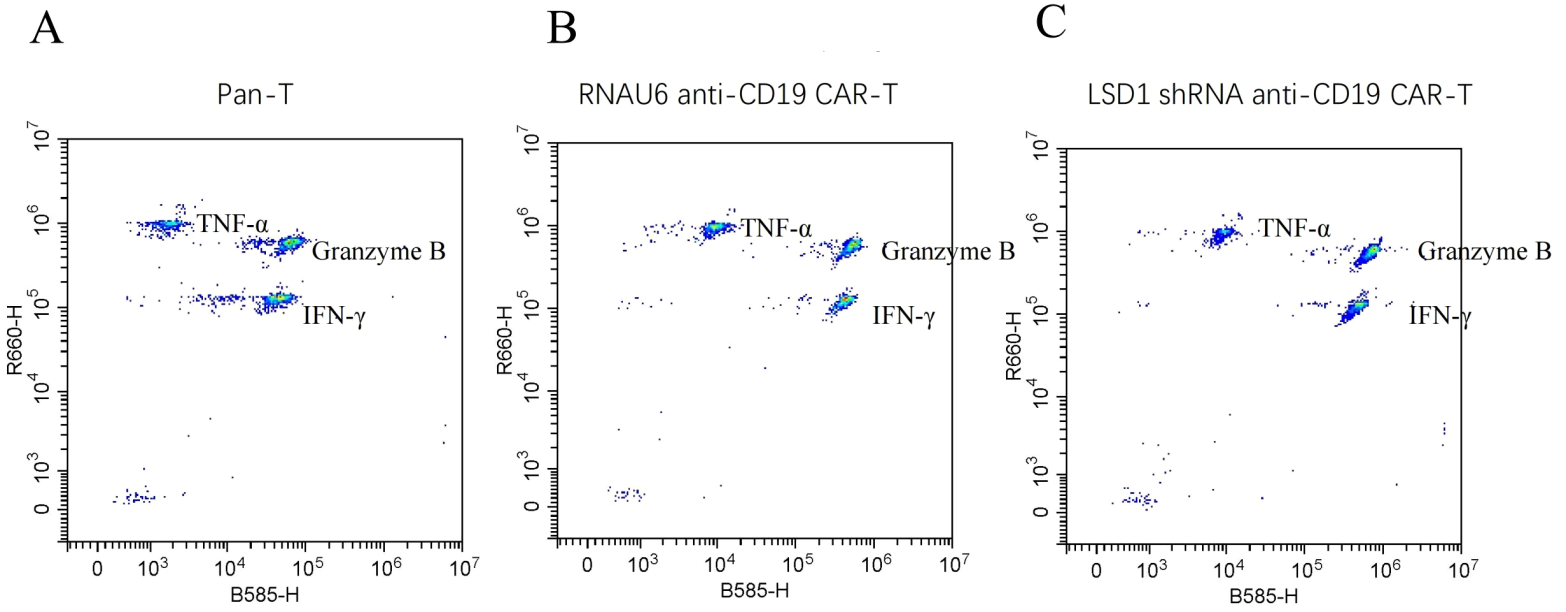
Representative images of cytokine secretion by flow cytometric analysis. **(A)** Peripheral blood mononuclear cell-derived T (pan-T) cells. **(B)** RNAU6 anti-CD19 CAR-T cells. **(C)** LSD1 shRNA anti-CD19 CAR-T cells.

To determine the killing efficiency of each type of CAR-T cell, we co-cultured RNAU6 anti-CD19 CAR-T cells or LSD1 shRNA anti-CD19 CAR-T cells with U-2932 cells (4:1, 2:1, 1:1, and 0.5:1) and assessed U-2932 apoptosis by flow cytometry after 16 h (**Figure 4A**). The early apoptosis and late apoptosis rates of the U-2932-Luc cells were significantly higher in the LSD1 shRNA anti-CD19 CAR-T co-culture group than in the RNAU6 anti-CD19 CAR-T group, indicative of superior killing efficiency by LSD1 shRNA anti-CD19 CAR-T cells (**Figures 4B, C**). We further found that LSD1 shRNA anti-CD19 CAR-T cells had comparable levels of TNF-α secretion to RNAU6 anti-CD19 CAR-T cells (**Figure 4D**), but secreted more IFN-γ, which is capable of indirectly killing tumor cells and affecting immune regulation. They also secreted more granzyme B, which is a cytotoxic molecule that can participate in cancer cell killing by T cells (**Figures 4E, F**). **Figure 5** shows a representative image of cytokine secretion analyzed by flow cytometry (**Figure 5**).

### 3.3 CD19-LSD1 shRNA CAR-T cells proliferated upon exposure to target cells

**Figure 6:**
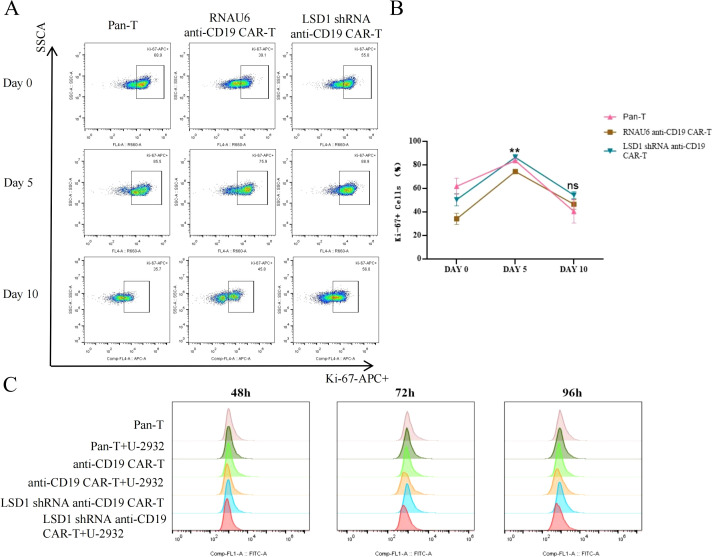
CAR-T cell *in vitro* proliferation assay. **(A)** Representative images of CAR-T cell proliferation (Ki-67^+^ cells) as detected by flow cytometry. **(B)** The proportion of Ki-67^+^ CAR-T cells. LSD1 shRNA anti-CD19 CAR-T cells were co-cultured with U-2932 cells over an effector target ratio gradient for 0, 5, or 10 d. The proliferating cells (Ki-67^+^) were measured by flow cytometry. Values are expressed as the mean ± standard deviation. Unpaired t-tests were performed between the groups. **p < 0.01 and ns, p > 0.05 compared with RNAU6 anti-CD19 CAR-T cells. **(C)** The proliferation of anti-CD19 CAR-T cells measured by the carboxyfluorescein diacetate succinimidyl ester method. The cells were stimulated by co-culture with U-2932 cells at an effector target ratio of 3:1. The assay was performed in the continuous presence of IL-2. The carboxyfluorescein diacetate succinimidyl ester signal is shown at 48, 72, and 96 h for each population shown.

After we co-cultured RNAU6 anti-CD19-CAR-T cells or LSD1 shRNA anti-CD19 CAR-T cells with U-2932 cells (2:1), we assessed CAR-T cell expansion by measuring Ki67 expression by flow cytometry after 0, 5, and 10 d (**Figures 6A, B**). On day 0, only a small percentage of CAR-T cells were Ki-67^+^; after U-2932 cell stimulation, CAR-T cells significantly proliferated and sustained that proliferation until day 10. We also co-cultured carboxyfluorescein succinimidyl ester-labeled CD19 CAR-T cells or LSD1 shRNA anti-CD19 CAR-T cells with U-2932 cells (3:1) and evaluated the changes in fluorescence of CAR-T cells by flow cytometry after 48, 72, and 96 h to statistically measure the expansion level. CD19 CAR-T and LSD1 shRNA anti-CD19 CAR-T cells both displayed greater proliferation after incubation with target cells for 72 and 96 h, and there was no statistical difference between CD19 CAR-T and LSD1 shRNA anti-CD19 CAR-T cell proliferation. The effect of LSD1 shRNA co-expression on the proliferative capacity of CD19 CAR-T may be more marked in *in vivo* experiments (**Figure 6C**).

### 3.4 CD19-LSD1 shRNA CAR-T cells developed a higher proportion of TCM than anti-CD19 CAR-T cells

**Figure 7:**
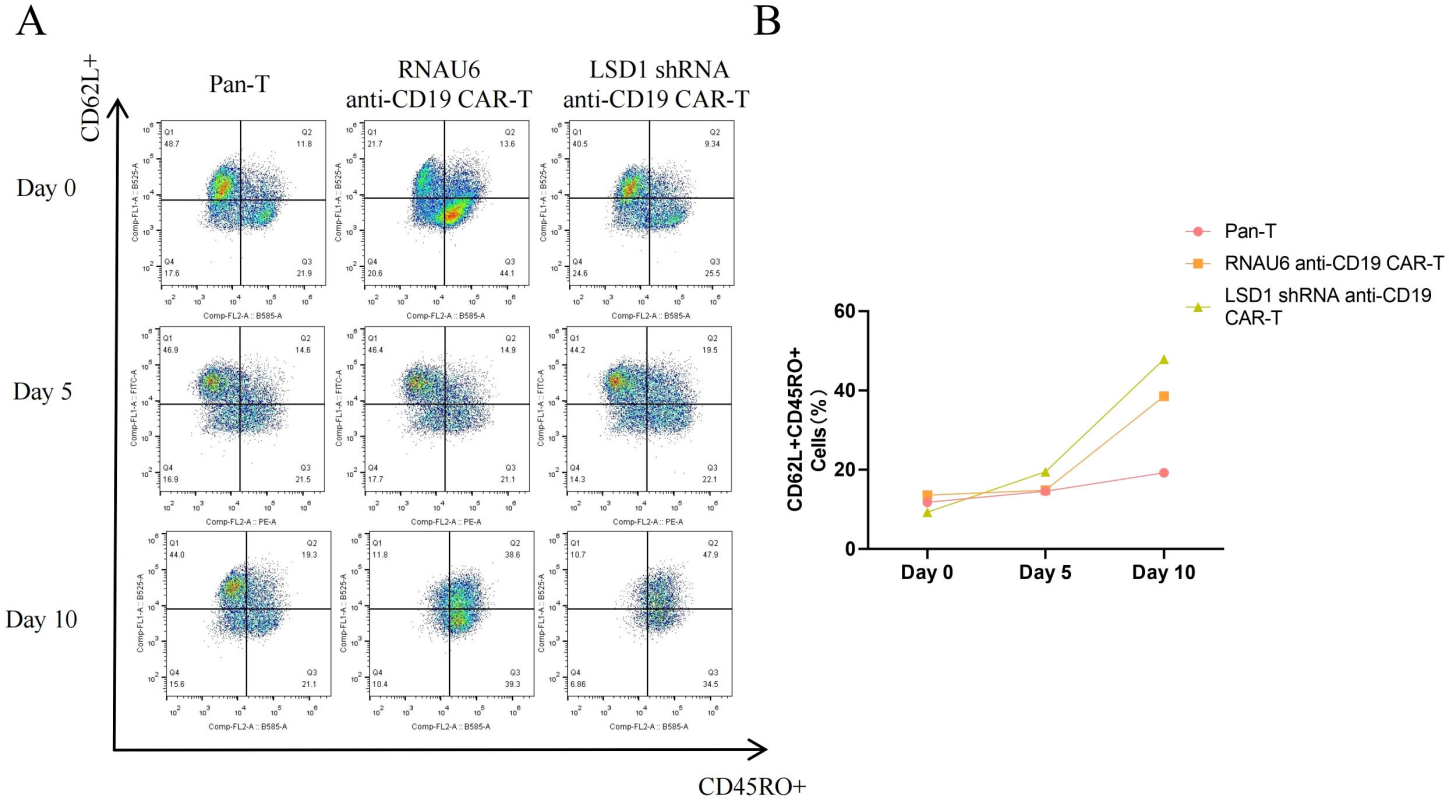
The proportion of memory T cells (CD62L^+^CD45RO^+^) among anti-CD19 CAR-T cells as measured by flow cytometry. **(A)** Representative flow cytometric profiles of memory T cells among CD3^+^ T cells. **(B)** The proportion of memory T cells.

TCM play an important role in CAR-T cell therapy, as they provide long-term immune memory and antigen-specific responses. CAR-T cell therapy uses genetic modification techniques to enable T cells to recognize and attack tumor cells. In long-term immunological memory responses, TCM respond rapidly and efficiently when they re-encounter their specific antigen, thereby enhancing the anti-tumor effects of CAR-T cells. In addition, a high percentage of TCM suggests that a therapeutic effect can be achieved with a very small infusion dose, further suggesting a favorable safety profile, which is critical to the success of CAR-T cell therapy. Therefore, we utilized flow cytometry to detect the percentage of TCM among the CAR-T cells. The percentage of cells with a TCM phenotype increased for both RNAU6 anti-CD19-CAR-T and LSD1 shRNA anti-CD19 CAR-T after stimulation via co-culture with U-2932 cells (**Figures 7A, B**). In conclusion, our results suggested that LSD1 downregulation enhances the function of CD19-specific CAR-T cells *in vitro*.

## 4 Discussion

CAR-T cell therapy, an effective method of immunotherapy for relapsed and refractory hematologic tumors, requires CAR-T cell preparation via the expansion of modified genetically engineered T cells (22). Clinical applications require monitoring of CAR-T cells to assess their cellular expansion and persistence *in vivo*. The proliferative ability and persistence of CAR-T cells *in vivo* can be monitored by flow and quantitative polymerase chain reaction assays. To an extent, the proliferative level determines the anti-tumor efficacy of CAR-T cells *in vivo* (23–25).

Manufacturing CAR-T cells involves the stimulation of single, nucleated cells enriched for T cells with antibody-encapsulated beads or plate-conjugated antibodies that induce T cell activation. This is followed by genetic modification using retroviral vectors or other delivery methods to facilitate the expression of CAR molecules on the cell surface (26–28). Reagents used for *in vitro* CAR-T cell expansion include culture media, serum, cytokines, and additional media supplements that collectively have a marked impact on CAR-T cell function. Moreover, specific conditions, such as the use of frozen, single, nucleated cells; T cell enrichment stimulation methods; and gene delivery procedures all affect CAR-T cell expansion. The choice of culture medium is the primary consideration for *in vitro* CAR-T cell expansion (29). Our choice, X-VIVO medium, has been widely used for T cell manufacturing. It is important to choose the right medium to expand sufficient numbers of T cells to fulfill research or clinical therapeutic needs (30, 31).

The generation of CAR-T cells requires the transfer of CAR genes into primary T cells, usually via γ-retroviral vectors. In this study, we enabled the stable expression of CAR molecules on the surface of T cells by infecting activated T cells with γ-retroviral vectors. The *in vitro* preparation of CAR-T cell products involves the collection and preparation of lymphocytes from patients, and encompasses a wide range of factors, such as the source of the T cells and the method of preparation. It also requires a complex evaluation of the products, entailing assessment of CAR-T cell viability, cell purity, microbial safety, and biological efficacy. A key aspect of CAR-T cell product quality control is the evaluation of target cell killing activity, which is closely related to efficacy. CAR-T cell production is a multi-step process that requires precise, safe management systems, including Good Manufacturing Practices and online quality control and assurance, to ensure and enhance the anti-tumor response and safety of the T cells. In this study, we successfully constructed a new cell therapy product via simultaneous expression of LSD1 shRNA and anti-CD19 CAR in human T cells, without affecting the expression of the CAR, by driving LSD1 shRNA expression under the U6 promoter and anti-CD19 CAR expression under the EF1a promoter. Current CAR-T cell therapy products under clinical evaluation are mainly second-generation CAR structures, which are limited by the short survival time of the CAR-T cells *in vivo* and relapse after effective CAR-T cell therapy (32). Our design provides a promising therapeutic approach for optimizing second-generation CAR structures and co-expressing novel shRNA-based regulatory factors to prevent T cell depletion. LSD1 shRNA overexpression enhanced the killing efficiency of CD19-specific CAR-T cells. Furthermore, the therapeutic efficacy and durability of the CAR-T cells significantly correlated with their differentiation status. The different stages of T cell differentiation include naïve T cells, stem cell central memory T cells, TCM, effector memory T cells, effector T cells, and terminally differentiated T cells. CAR-T cell products with high levels of naïve T cells, TCM, and stem cell central memory T cells have shown excellent anti-tumor responses and *in vivo* persistence (33). Several clinical T cell trials have reported a positive correlation between infusion of less differentiated T cells and better clinical outcomes; thus, focusing on memory cell formation is one of the recent strategies to improve CAR-T cell persistence (34). In this study, co-expression of LSD1 shRNA in anti-CD19 CAR- T cells resulted in a higher percentage of CAR-T cells with the TCM phenotype. Elevated levels of TCM cells may be related to the long-term persistence of T cells, which could further enhance the effectiveness of CAR-T cell therapy.

Although CAR-T cell therapy is an effective immunotherapy for the treatment of relapsed and refractory hematologic tumors, its success remains limited by poor *in vivo* expansion of CAR-T cells, relapse after CAR-T cell therapy, and toxicity problems. In a long-term follow-up of 101 patients with relapsed and refractory DLBCL treated with tisagenlecleucel, 61 patients experienced disease progression or died during the study period. The median progression-free survival time was only 5.9 months (35). Therefore, it is important to reconsider the structure of CAR-T cells, including the ecto-structural domains, transmembrane structural domains, and endo-structural domains, and the cell preparation techniques and T cell sources, to improve the clinical efficiency, persistence, infiltration, and resistance to apoptosis of CAR-T cells (36). CARs are synthetic cell-surface receptors that redirect cytotoxic immune cells to target cells expressing cell-surface antigens independent of major histocompatibility complex molecules. The primary function of a CAR is to replace the T cell receptor–CD3 complex and deliver the initiating signals for T cell activation. CARs usually consist of four structural domains: an extracellular antigen-binding structural domain, a spacer or hinge region, a transmembrane structural domain, and an intracellular signaling structural domain (37). CAR structures have evolved over time and have now been upgraded to a fourth generation. The first-generation CAR structure has only one intracellular CD3ζ signaling domain; although it can exert certain anti-tumor activity, it cannot transduce proliferative signals or induce the production of a large number of cytokines, which makes the downstream killing effect on tumors unsatisfactory. The second-generation CAR added an intracellular co-stimulatory molecule, such as CD28/B7, ICOS, 4-1BB (CD137), CD27, and OX40 (CD134). The co-stimulatory molecule promotes the secretion of cytokines such as IL-2 by CAR-T cells, thereby increasing the vitality of T cells and prolonging T cell survival time. The third-generation CAR is based on the second-generation with the addition of a co-stimulatory molecule, mainly CD28 and CD137; it confers stronger cytokine release and tumor-killing ability, but is also prone to promoting the phenomenon of activation-induced cell death. Fourth-generation CARs, also known as “armored CARs,” are structurally different from the first three generations. The introduction of pro-inflammatory cytokines (currently mainly IL-12) and co-stimulatory ligands (4-1BBL and CD40L) has reduced the incidence of adverse effects during pretreatment therapy and elicited a wider range of anti-tumor immune effects (38).

**Figure 8:**
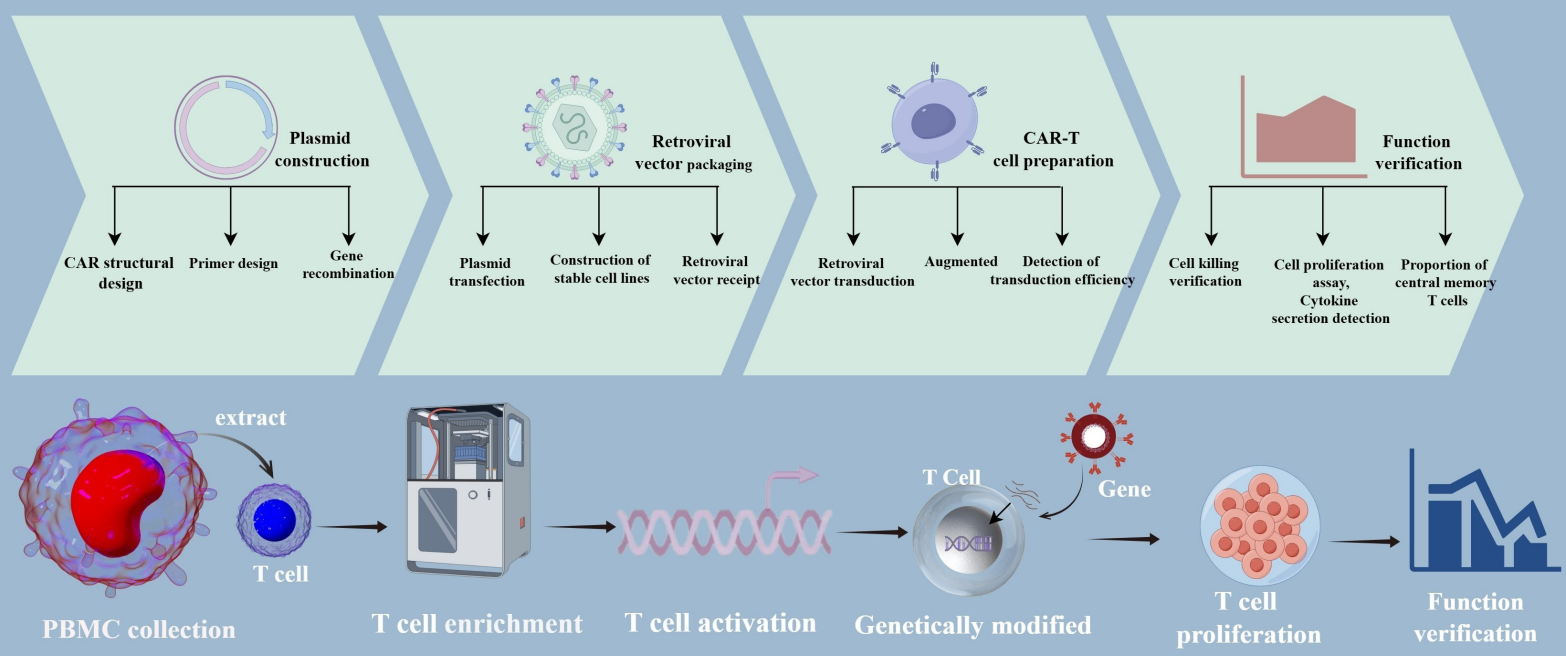
Diagram of the overall technical approach.

LSD1 is overexpressed in several cancer types, and its aberrant overexpression plays a critical role in cancer development and progression. Several LSD1 inhibitors developed to date effectively attenuate tumor growth both *in vivo* and *in vitro*. LSD1 is involved in anti-tumor immunity, and LSD1 inhibition enhances the responsiveness of CD8^+^ T cells, thereby improving the efficacy of T cell-based therapies (39). We found that overexpression of LSD1 shRNA enhanced the function of CD19-specific CAR-T cells and increased the killing efficiency of LSD1 shRNA anti-CD19 CAR-T cells compared with control CAR-T cells. Moreover, the efficacy and durability of the CAR-T cells were closely related to their differentiation status. The longevity, persistence, and functionality of T cells are key determinants of T cell-based therapies, and the phenotype of the T cell product can have a profound impact on treatment outcomes. Keeping T cells in a less differentiated state and maintaining their plasticity and adaptability after *in vitro* production and infusion may result in a stronger therapeutic effect (40). In this study, co-expression of shLSD1 in anti-CD19 CAR- T cells resulted in an increase in the proportion of CAR-T cells with a TCM phenotype. Elevated proportions of TCM may be associated with the long-term persistence of T cells, which could further enhance the effectiveness of CAR-T cell therapy. This is consistent with the finding that LSD1 inhibitor-treated CAR-T cells showed a less differentiated phenotype, with an increased proportion of TCM and similar initial T cells, along with a decreased proportion of terminal effector T cells. The same study found that LSD1 enhanced the expression of the CAR and levels of HLA-DR after co-culture with tumor cells, suggesting enhanced tumor-specific anti-tumor function (41). The overall technological approach used in this study is illustrated in **Figure 8**. Although our study yielded encouraging results in *in vitro* experiments, the *in vivo* safety of the approach remains to be confirmed. In addition, the mechanism by which LSD1 shRNA enhances the functionality of CAR-T cells needs to be further explored. More research is needed to deepen our understanding of this strategy to better utilize its clinical value.

## Data Availability

The original contributions presented in the study are included in the article/supplementary material. Further inquiries can be directed to the corresponding author.

## References

[1] Salter AI Pont MJ Riddell SR. Chimeric antigen receptor-modified T cells: CD19 and the road beyond. Blood. (2018) 131:2621–9. doi: 10.1182/blood-2018-01-785840 PMC6032892 29728402

[2] Ying Z Huang XF Xiang X Liu Y Kang X Song Y. A safe and potent anti-CD19 CAR T cell therapy. Nat Med. (2019) 25:947–53. doi: 10.1038/s41591-019-0421-7 PMC7518381 31011207

[3] Denlinger N Bond D Jaglowski S. CAR T-cell therapy for B-cell lymphoma. Curr Probl Cancer. (2022) 46:100826. doi: 10.1016/j.currproblcancer.2021.100826 35012754 PMC9284423

[4] Boardman AP Salles G. CAR T-cell therapy in large B cell lymphoma. Hematol Oncol. (2023) 41 Suppl 1:112–8. doi: 10.1002/hon.31531 PMC10348487 37294963

[5] Alarcon Tomas A Fein JA Fried S Flynn JR Devlin SM Fingrut WB. Outcomes of first therapy after CD19-CAR-T treatment failure in large B-cell lymphoma. Leukemia. (2023) 37:154–63. doi: 10.1038/s41375-022-01739-2 PMC9892211 36335261

[6] Zhong N Ma Q Gong S Shi Y Zhao L Wang D. Rapid response in relapsed follicular lymphoma to novel anti-CD19 CAR-T therapy with pseudo-progression and cytomegalovirus infection: A case report. Int Immunopharmacol. (2024) 134:112174. doi: 10.1016/j.intimp.2024.112174 38703571

[7] Lutfi F Patel A Mehta J Goyal A Dahiya S. Second-line treatment with CAR T-cell therapy for large B-cell lymphoma. Clin Adv Hematol Oncol. (2023) 21:170–8. 37039724

[8] Gideon J. CAR T-cell therapy for relapsed/refractory aggressive large B-cell lymphoma. Clin J Oncol Nurs. (2022) 26:597–601. doi: 10.1188/22.CJON.597-601 36413724

[9] Neelapu SS Locke FL Bartlett NL Lekakis LJ Miklos DB Jacobson CA. Axicabtagene ciloleucel CAR T-cell therapy in refractory large B-cell lymphoma. N Engl J Med. (2017) 377:2531–44. doi: 10.1056/NEJMoa1707447 PMC5882485 29226797

[10] Westin JR Oluwole OO Kersten MJ Miklos DB Perales MA Ghobadi A. Survival with axicabtagene ciloleucel in large B-cell lymphoma. N Engl J Med. (2023) 389:148–57. doi: 10.1056/NEJMoa2301665 37272527

[11] Locke FL Miklos DB Jacobson CA Perales MA Kersten MJ Oluwole OO. Axicabtagene ciloleucel as second-line therapy for large B-cell lymphoma. N Engl J Med. (2022) 386:640–54. doi: 10.1056/NEJMoa2116133 34891224

[12] Dreger P Corradini P Gribben JG Glass B Jerkeman M Kersten MJ. CD19-directed CAR T cells as first salvage therapy for large B-cell lymphoma: towards a rational approach. Lancet Haematol. (2023) 10:e1006–e15. doi: 10.1016/S2352-3026(23)00307-1 38030311

[13] Brudno JN Lam N Vanasse D Shen YW Rose JJ Rossi J. Safety and feasibility of anti-CD19 CAR T cells with fully human binding domains in patients with B-cell lymphoma. Nat Med. (2020) 26:270–80. doi: 10.1038/s41591-019-0737-3 PMC7781235 31959992

[14] Chihara D Liao L Tkacz J Franco A Lewing B Kilgore KM. Real-world experience of CAR T-cell therapy in older patients with relapsed/refractory diffuse large B-cell lymphoma. Blood. (2023) 142:1047–55. doi: 10.1182/blood.2023020197 37339585

[15] Zhang X Zhu L Zhang H Chen S Xiao Y. CAR-T cell therapy in hematological Malignancies: current opportunities and challenges. Front Immunol. (2022) 13:927153. doi: 10.3389/fimmu.2022.927153 35757715 PMC9226391

[16] Belk JA Daniel B Satpathy AT. Epigenetic regulation of T cell exhaustion. Nat Immunol. (2022) 23:848–60. doi: 10.1038/s41590-022-01224-z PMC10439681 35624210

[17] Dai XJ Liu Y Xiong XP Xue LP Zheng YC Liu HM. Tranylcypromine based lysine-specific demethylase 1 inhibitor: summary and perspective. J Med Chem. (2020) 63:14197–215. doi: 10.1021/acs.jmedchem.0c00919 32931269

[18] Fang Y Liao G Yu B. LSD1/KDM1A inhibitors in clinical trials: advances and prospects. J Hematol Oncol. (2019) 12:129. doi: 10.1186/s13045-019-0811-9 31801559 PMC6894138

[19] Qiu F Jiang P Zhang G An J Ruan K Lyu X. Priming with LSD1 inhibitors promotes the persistence and antitumor effect of adoptively transferred T cells. Nat Commun. (2024) 15:4327. doi: 10.1038/s41467-024-48607-4 38773088 PMC11109160

[20] Sheng W Liu Y Chakraborty D Debo B Shi Y. Simultaneous inhibition of LSD1 and TGFβ Enables eradication of poorly immunogenic tumors with anti-PD-1 treatment. Cancer Discovery. (2021) 11:1970–81. doi: 10.1158/2159-8290.CD-20-0017 PMC8598400 33687985

[21] Sheng W LaFleur MW Nguyen TH Chen S Chakravarthy A Conway JR. LSD1 ablation stimulates anti-tumor immunity and enables checkpoint blockade. Cell. (2018) 174:549–63.e19. doi: 10.1016/j.cell.2018.05.052 29937226 PMC6063761

[22] Vandghanooni S Eskandani M Sanaat Z Omidi Y. Recent advances in the production, reprogramming, and application of CAR-T cells for treating hematological Malignancies. Life Sci. (2022) 309:121016. doi: 10.1016/j.lfs.2022.121016 36179813

[23] Kunz A Gern U Schmitt A Neuber B Wang L Hückelhoven-Krauss A. Optimized assessment of qPCR-based vector copy numbers as a safety parameter for GMP-grade CAR T cells and monitoring of frequency in patients. Mol Ther Methods Clin Dev. (2020) 17:448–54. doi: 10.1016/j.omtm.2020.02.003 PMC7078460 32201711

[24] Schubert ML Kunz A Schmitt A Neuber B Wang L Hückelhoven-Krauss A. Assessment of CAR T cell frequencies in axicabtagene ciloleucel and tisagenlecleucel patients using duplex quantitative PCR. Cancers (Basel). (2020) 12:2820. doi: 10.3390/cancers12102820 33007926 PMC7601213

[25] McLellan AD Ali Hosseini Rad SM. Chimeric antigen receptor T cell persistence and memory cell formation. Immunol Cell Biol. (2019) 97:664–74. doi: 10.1111/imcb.12254 31009109

[26] Poorebrahim M Quiros-Fernandez I Fakhr E Cid-Arregui A. Generation of CAR-T cells using lentiviral vectors. Methods Cell Biol. (2022) 167:39–69. doi: 10.1016/bs.mcb.2021.07.001 35152998

[27] Watanabe N McKenna MK. Generation of CAR T-cells using γ-retroviral vector. Methods Cell Biol. (2022) 167:171–83. doi: 10.1016/bs.mcb.2021.06.014 PMC8917789 35152995

[28] Mo F Mamonkin M. Generation of chimeric antigen receptor T cells using gammaretroviral vectors. Methods Mol Biol. (2020) 2086:119–30. doi: 10.1007/978-1-0716-0146-4_8 31707671

[29] Stock S Schmitt M Sellner L. Optimizing manufacturing protocols of chimeric antigen receptor T cells for improved anticancer immunotherapy. Int J Mol Sci. (2019) 20:6223. doi: 10.3390/ijms20246223 31835562 PMC6940894

[30] Koch E Hopmann C Fröhlich LF Schebb NH. Fatty acid and oxylipin concentration differ markedly between different fetal bovine serums: A cautionary note. Lipids. (2021) 56:613–6. doi: 10.1002/lipd.12321 34435366

[31] Watanabe N Mo F McKenna MK. Impact of manufacturing procedures on CAR T cell functionality. Front Immunol. (2022) 13:876339. doi: 10.3389/fimmu.2022.876339 35493513 PMC9043864

[32] Wei J Guo Y Wang Y Wu Z Bo J Zhang B. Clinical development of CAR T cell therapy in China: 2020 update. Cell Mol Immunol. (2021) 18:792–804. doi: 10.1038/s41423-020-00555-x 32999455 PMC8115146

[33] Lim WA June CH. The principles of engineering immune cells to treat cancer. Cell. (2017) 168:724–40. doi: 10.1016/j.cell.2017.01.016 PMC5553442 28187291

[34] Jafarzadeh L Masoumi E Fallah-Mehrjardi K Mirzaei HR Hadjati J. Prolonged persistence of chimeric antigen receptor (CAR) T cell in adoptive cancer immunotherapy: challenges and ways forward. Front Immunol. (2020) 11:702. doi: 10.3389/fimmu.2020.00702 32391013 PMC7188834

[35] Jaeger U Tam CS Borchmann P McGuirk JP Johansen M Waller EK. Long-term safety for patients with tisagenlecleucel-treated relapsed/refractory diffuse large B-cell lymphoma. Blood Adv. (2022) 6:4816–20. doi: 10.1182/bloodadvances.2021006193 PMC9631665 35687492

[36] Huang R Li X He Y Zhu W Gao L Liu Y. Recent advances in CAR-T cell engineering. J Hematol Oncol. (2020) 13:86. doi: 10.1186/s13045-020-00910-5 32616000 PMC7333410

[37] Pan K Farrukh H Chittepu V Xu H Pan CX Zhu Z. CAR race to cancer immunotherapy: from CAR T, CAR NK to CAR macrophage therapy. J Exp Clin Cancer Res. (2022) 41:119. doi: 10.1186/s13046-022-02327-z 35361234 PMC8969382

[38] Jayaraman J Mellody MP Hou AJ Desai RP Fung AW Pham AHT. CAR-T design: Elements and their synergistic function. EBioMedicine. (2020) 58:102931. doi: 10.1016/j.ebiom.2020.102931 32739874 PMC7393540

[39] Dong J Pervaiz W Tayyab B Li D Kang L Zhang H. A comprehensive comparative study on LSD1 in different cancers and tumor specific LSD1 inhibitors. Eur J Med Chem. (2022) 240:114564. doi: 10.1016/j.ejmech.2022.114564 35820351

[40] Mondino A Manzo T. To remember or to forget: the role of good and bad memories in adoptive T cell therapy for tumors. Front Immunol. (2020) 11:1915. doi: 10.3389/fimmu.2020.01915 32973794 PMC7481451

[41] Pallavicini I Frasconi TM Catozzi C Ceccacci E Tiberti S Haas D. LSD1 inhibition improves efficacy of adoptive T cell therapy by enhancing CD8(+) T cell responsiveness. Nat Commun. (2024) 15:7366. doi: 10.1038/s41467-024-51500-9 39191730 PMC11349769

